# Metal ions from S‐PRG filler have the potential to prevent periodontal disease

**DOI:** 10.1002/cre2.70

**Published:** 2017-08-10

**Authors:** Yoko Iwamatsu‐Kobayashi, Syouta Abe, Yoshiyasu Fujieda, Ai Orimoto, Masafumi Kanehira, Keisuke Handa, Venkata Suresh Venkataiah, Wei Zou, Masaki Ishikawa, Masahiro Saito

**Affiliations:** ^1^ Division of Operative Dentistry, Department of Restorative Dentistry Tohoku University Graduate School of Dentistry Sendai Miyagi Japan; ^2^ Faculty of Industrial Science and Technology Tokyo University of Science Katsushika Japan; ^3^ Center for Advanced Stem Cell and Regenerative Research Tohoku University Graduate School of Dentistry Sendai Miyagi Japan

**Keywords:** periodontal disease, bone loss, inflammation, S‐PRG filler

## Abstract

The surface pre‐reacted glass ionomer (S‐PRG) filler, a component of composite resin, is capable of releasing metal ions that possess antibacterial activity against caries and periodontal pathogens. Although S‐PRG has been suggested to be involved in oral disease prevention, no reports have been published regarding its preventive effect on periodontal disease *in vivo*. The present study investigated whether the eluate from S‐PRG (S‐PRG eluate) has a suppressive effect on tissue destruction induced in a mouse model of ligature‐induced periodontal disease. Twenty‐seven C57BL/6 mice were divided into three groups of nine animals each, no ligature group (Lig(−)), ligature group (Lig(+)S‐PRG(−)) and ligature with S‐PRG eluate group (Lig(+)S‐PRG(+)). Alveolar bone loss was evaluated using micro‐computed tomography scanning. Histologic changes were detected by hematoxylin and eosin staining. The infiltration of inflammatory cells was assessed by Ly6G and F4/80 staining immunohistochemically. The distribution of metal ions was detected by time‐of‐flight secondary ion mass spectrometry. S‐PRG eluate clearly inhibited alveolar bone loss and bone density. The histological analysis revealed that S‐PRG eluate reduced destruction of the collagen bundle in the periodontal ligament and the infiltration of inflammatory cells. Immunohistochemical analysis showed that the S‐PRG eluate significantly suppressed the number of infiltrating neutrophils and macrophages. Time‐of‐flight secondary ion mass spectrometry analysis revealed that more boron ions were present in the Lig(+)S‐PRG(+) group than in the Lig(+)S‐PRG(−) group. Our results suggest that the S‐PRG eluate has a preventive effect against tissue destruction in periodontal disease through its anti‐inflammatory effects *in vivo*.

## 1 INTRODUCTION

Periodontal diseases are characterized by progressive inflammation‐induced alveolar bone loss resulting from an altered host–biofilm interaction (Sima & Glogauer, 2013; Yu et al., 2016). Acute inflammation is a rapid process characterized by fluid exudation and emigration of leukocytes, primarily neutrophils, whereas chronic inflammation extends over a longer time period and is associated with lymphocyte and macrophage infiltration (Hasturk, Kantarci, & Van Dyke, 2012). It has been reported that neutrophil extracellular traps in periodontitis represent a novel paradigm in neutrophil‐mediated immunity, and macrophage depletion abates *Porphyromonas gingivalis*‐induced alveolar bone resorption in mice (Eskan et al., 2012; Hajishengallis & Sahingur, 2014; Lam et al., 2014).

Traditional therapeutic approaches for periodontal disease include the mechanical removal of the bacterial biofilm with conventional periodontal and/or surgical treatments, and various chemotherapeutic agents have been shown to be efficacious for the treatment of periodontal disease (Mombelli, Nyman, Bragger, Wennstrom, & Lang, 1995; van der Ouderaa, 1991). Recently, the bactericidal activity of materials coated with metal and metalloid ions has led to the suggestion that they may be used as novel anti‐bacterial therapeutics against periodontal bacteria, as well as antibiotics (Goudouri, Kontonasaki, Lohbauer, & Boccaccini, 2014). A surface pre‐reacted glass ionomer (S‐PRG) filler is able to release six ions—fluoride (F), strontium (Sr), sodium (Na), boron (B), aluminum (Al), and silica (Si)—and has been shown to possess properties such as the prevention of demineralization, promotion of remineralization, and bactericidal activity (Fujimoto et al., 2010; Han & Okiji, 2011; Hosoya et al., 2013; Miki et al., 2016; Murayama et al., 2012; Saku, et al., 2010). It has been reported that S‐PRG eluate inhibits the protease and gelatinase activities of *P. gingivalis* and suppresses co‐aggregation between *P. gingivalis* and *Fusobacterium nucleatum* (Yoneda et al., 2012). It also inhibits biofilm formation and disrupts mature biofilms (Shimazu, Oguchi, Takahashi, Konishi, & Karibe, 2015; Suzuki, et al., 2014). These findings indicate that S‐PRG could be useful as a preventive dental material in periodontal disease. However, there are no reports regarding the effect of S‐PRG filler on periodontitis *in vivo*.

In the present study, we hypothesized that the S‐PRG eluate has the potential to prevent the progression of periodontal disease. To explore this hypothesis, we used the mouse model of ligature‐induced periodontal disease to investigate the effects of the S‐PRG eluate on periodontal tissue destruction.

## 2 MATERIALS AND METHODS

### 2.1 Animals

Twenty‐seven specific‐pathogen‐free female C57BL/6 mice were purchased from the Kumagai Laboratory (Sendai, Japan). All mouse experimental procedure described in this study was reviewed and approved by the Tohoku University Institutional Animal Care and Use Committee. Each group of mice was maintained in individual ventilated cages and provided sterile food and water under specific‐pathogen‐free conditions.

### 2.2 Experimental periodontal disease model

Periodontal disease was experimentally induced to the mice as described by Abe et al. (Abe & Hajishengallis, 2013). Briefly, the mice were given general anesthesia of 0.3 mg/kg of medetomidine, 4 mg/kg of midazolam, and 5 mg/kg of butorphanol (intraperitoneally). The ligature‐induced periodontal disease model was created by inserting a 5‐0 silk ligature (Ethicon, NJ, USA) around the mandibular right second molar. Suture was applied and tied gently to prevent damage to the periodontal tissue.

### 2.3 Preparation of the S‐PRG eluate

S‐PRG eluate was prepared according to the method described by Fujimoto et al. (Fujimoto et al., 2010). Briefly, S‐PRG filler (SHOFU Inc., Kyoto, Japan) was mixed with an equal amount of distilled water and shaken gently at room temperature for 24 hr. The filler material was removed by filtration, and the ion solution was centrifuged to remove any residual insoluble material. The clear supernatant was collected and used as the S‐PRG eluate. The ion concentration of S‐PRG was as follows: Al, 12.1 ppm; B, 2001.1 ppm; Na, 544.5 ppm; Si, 14.0 ppm; Sr, 103.5 ppm; and F, 110.5 ppm. We used this eluate throughout the manuscript.

### 2.4 Experimental design

**Figure 1:**
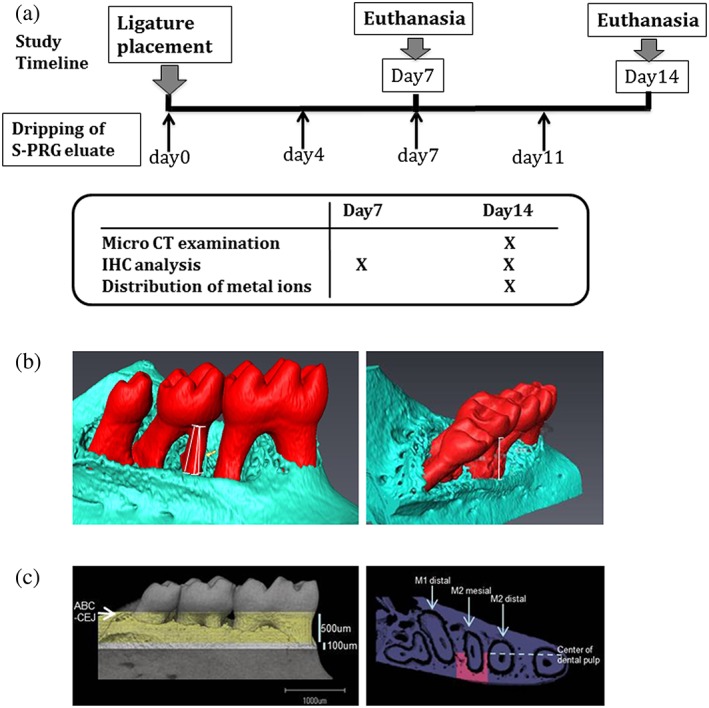
Study timeline and three‐dimensional measurements in the analysis of tooth‐supporting alveolar bone. (a) The study timeline showing the time at which the surface pre‐reacted glass ionomer (S‐PRG) eluate was administered after ligature placement. Only immunohistochemical analysis was performed on day 7 and day 14. (b) A three‐dimensional view of the mandible at the site of ligature placement. Linear bone loss between the cementoenamel junction and alveolar bone crest was measured along the mesial root of M2. (c) The bone volume fraction and density around the M2 mesial root were measured in the area between 500 and 600 μm from the cementum enamel junction‐alveolar bone crest, between the distal margin of the M1 distal root and the mesial margin of the M2 distal root, and between the alveolar bone surface and the center of the dental pulp of the M2 mesial root

At 8 weeks of age, the animals were randomly assigned to three experimental groups of nine mice each: no ligature group (Lig(−)), ligature group (Lig(+)S‐PRG(−)) or ligature with 10 μL S‐PRG eluate dripped onto the 5‐0 silk on days 0, 4, 7, and 11 after ligature application group (Lig(+)S‐PRG(+)) (Figure 1a). All the experiments were performed three times and confirmed reproducible.

### 2.5 Sample collection

Eighteen mice were euthanized on day 14 for all experiments (*N* = 6 each). Nine mice were also euthanized on day 7 only for immunohistochemical (IHC) analysis (*N* = 3 each). The mandibular bone specimens were harvested and fixed in 4% paraformaldehyde for 1 day, and transferred to phosphate‐buffered saline (PBS).

### 2.6 Micro‐computed tomography examination for alveolar bone loss

Fixed right mandibles of Lig(−), Lig(+)S‐PRG(−), and Lig(+)S‐PRG(+) on day 14 (*N* = 6 each) were removed and scanned using a cone beam micro‐computed tomography (micro‐CT) system at Kureha Special Laboratory (Iwaki, Japan). The X‐ray generator was operated at an acceleration potential of 75 kV with a beam current of 100 mA. The scans were reconstructed, and three‐dimensional digitized images were generated for each specimen. Using Avizo software (Maxnet, Tokyo, Japan), each reconstructed image was rotated into a standardized orientation. To assess linear alveolar bone loss, the cementum enamel junction‐alveolar bone crest (CEJ‐ABC) distances were measured at three sites in each root of the lingual surfaces of the ligated side. The bone volume fraction and density around the mesial root of the second molar (M2) were measured in the area between 500 and 600 μm from the CEJ‐ABC, between the distal margin of the first molar (M1) distal root and the mesial margin of the M2 distal root, and between the alveolar bone surface and the center of the dental pulp of the M2 mesial root (Figure 1b,c).

### 2.7 Histological and IHC examination

Histological and IHC examinations were performed using right mandibles at day 7 (*N* = 3 each) and day 14 (*N* = 3 each). In the case of day 14, samples were obtained after micro‐CT analysis. The specimens were decalcified with 10% EDTA, embedded in paraffin, and cut into 5‐μm‐thick serial sagittal sections. The sections were stained with hematoxylin and eosin. For IHC examination, the sections were deparaffinized and rehydrated with graded ethanol. Further digestion for antigen retrieval was performed using 0.1% pepsin in 0.01 N hydrochloric acid at room temperature (RT) for 30 min. Nonspecific binding was blocked by incubation in PBS containing 5% skim milk for 1 hr at RT. The sections were then incubated with primary antibodies at 4°C overnight. After washing with PBS containing 0.5% skim milk, the sections were incubated with the secondary antibodies at RT for 1 hr. The primary antibodies were rat anti‐Ly6G (Abcam, Cambridge, UK) and rat anti‐F4/80 (Abcam). The secondary antibody was Alexa Fluor 555 donkey anti‐rat IgG (Life Technologies, Grand Island, NY, USA), and nuclear staining was performed with Hoechst 33342 (Life Technologies). Histological and IHC images were obtained with a Leica CTR6000 microscope. The fluorescence intensities were quantified with ImageJ software (National Institutes of Health, Bethesda, MD, USA).

### 2.8 Distribution of metal ions in histological specimens

Mandibular bone specimens after micro‐CT analysis (*N* = 3 each) were dehydrated in an ascending series of alcohol and xylene without decalcification and then embedded with the Osteoresin embedding kit (Wako Pure Chemical Industries, Osaka, Japan). Sagittal sections of the embedded specimens were mirror‐polished with waterproof abrasive paper. Time‐of‐flight secondary ion mass spectrometry (TOF‐SIMS; TOF‐SIMS V, ION‐TOF GmbH, Munster, Germany) was performed using a spectrometer equipped with a pulsed Bi+ liquid ion gun operated at 25 kV. The system vacuum was held below 10^−6^ Pa throughout the measurements. The average primary ion current was 1.25 pA, and the images were acquired in an area of 500 × 500 μm.

### 2.9 Statistical analysis

All values in this study were presented as mean ± SD. The statistical analysis was performed using SPSS software (V 21.0 for Windows, SPSS Inc., Chicago, IL, USA). Parameters were analyzed statistically using the Kruskal–Wallis test followed by Tukey's test. Statistically significant were considered at *p* < .05.

## 3 RESULTS

### 3.1 Effect of the S‐PRG eluate on alveolar bone loss

**Figure 2:**
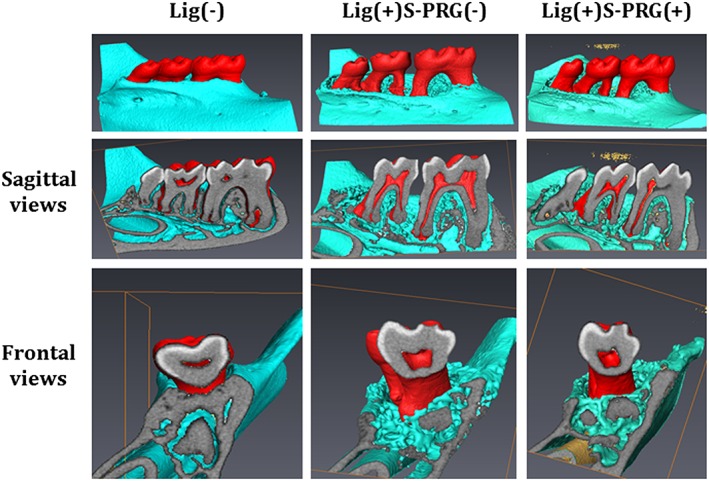
Representative micro‐computed tomography (micro‐CT) images of mandibular alveolar bone surrounding the second molars on day 14 are shown. Three‐dimensional (upper panel) and bidimensional sagittal (middle panel) and frontal (lower panel) micro‐CT views of mice in the Lig(−), Lig(+)S‐PRG(−), and Lig(+)S‐PRG(+) groups are provided to show the differences in bone resorption between the groups. Teeth, bone, and cross‐sectional surfaces are indicated by red, green, and gray, respectively

**Figure 3:**
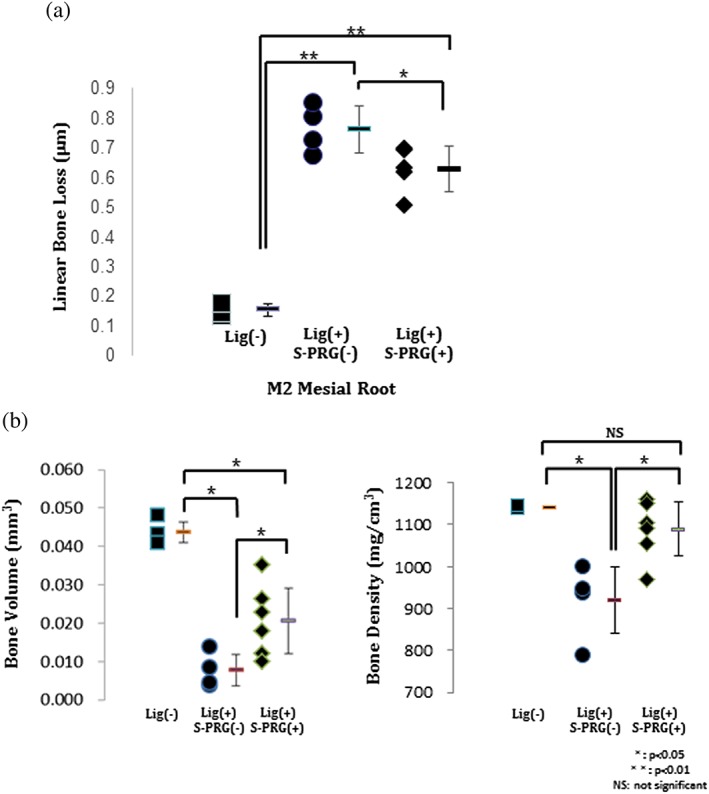
(a) Results of the quantitative analysis of the cementum enamel junction‐alveolar bone crest distance on day 14 after ligature placement. (b) Quantitative analysis of the bone volume and bone density of each group (*p < .05, **p < .01; NS = not significant)

To analyze the effect of the S‐PRG eluate in the periodontal disease model, alveolar bone loss was assessed by micro‐CT analysis. The three‐dimensional, bi‐dimensional sagittal, and frontal micro‐CT views (Figure 2) and overall view (Supplemental Videos S1 and S2) of the mandibular molars showed that alveolar bone loss around the M2 mesial root of Lig(+)S‐PRG(+) group was reduced compared to the Lig(+)S‐PRG(−) group. To quantitatively analyze alveolar bone loss, we measured the CEJ‐ABC distances in the M2 mesial root of the lingual surfaces of the mice (Figure 3a). The ABC‐CEJ distance in the M2 mesial root was 0.15 ± 0.02 mm in the Lig(−) group. By contrast, they were 0.76 ± 0.08 mm in the Lig(+)S‐PRG(−) group and 0.63 ± 0.08 mm in the Lig(+)S‐PRG(+) group. The ABC‐CEJ distance in the Lig(+)S‐PRG(+) group was significantly lower than that in the Lig(+)S‐PRG(−) group (*p* < .05).

The bone volume fraction was 0.044 ± 0.003 mm^3^ in the Lig(−) group, 0.008 ± 0.004 mm^3^ in the Lig(+)S‐PRG(−) group and 0.020 ± 0.009 mm^3^ in the Lig(+)S‐PRG(+) group. The bone density was 1,141.25 ± 3.00 mg/cm^3^ in the Lig(−) group, 921.40 ± 79.50 mg/cm^3^ in the Lig(+)S‐PRG(−) group, and 1,089.33 ± 63.91 mg/cm^3^ in the Lig(+)S‐PRG(+) group. The bone volume in the Lig(+)S‐PRG(+) group was significantly less than in the Lig(−) group but was more than in the Lig(+)S‐PRG(−) group. The bone density in the Lig(+)S‐PRG(+) group was also more than the Lig(+)S‐PRG(−). In addition, there was no significant difference in the bone density between the Lig(+)S‐PRG(+) group and the Lig(−) group (Figure 3b).

### 3.2 S‐PRG eluate prevents the inflammatory response

**Figure 4:**
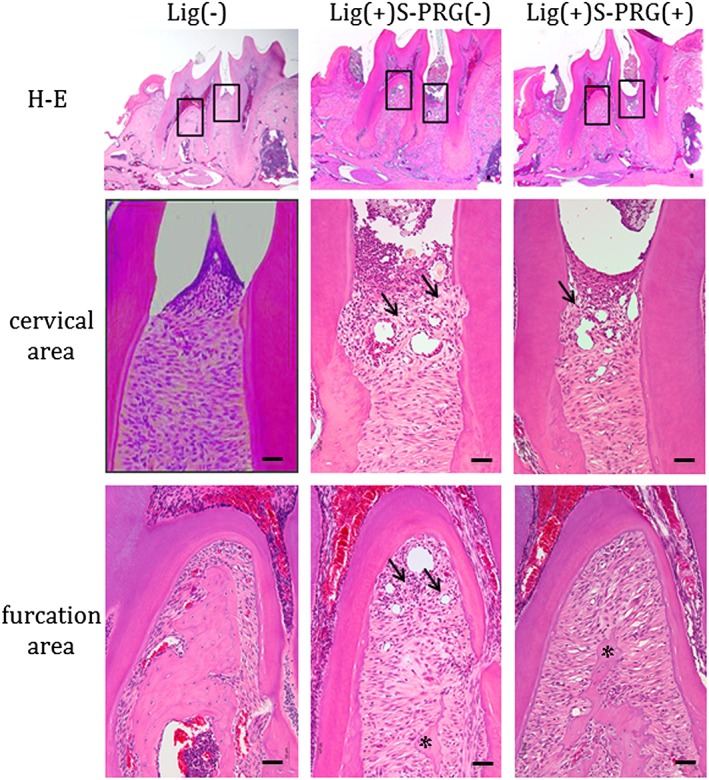
Results of the histological analysis of hematoxylin and eosin (H&E) staining of day 14 samples are shown (upper panel). The squares indicate the areas that are presented at a higher magnification in the center (cervical) and bottom (furcation) rows (bar: 50 μm). Arrows showed collagen bundle destruction and asterisks showed bone resorption

Histological average images were shown in Figure 4. The results indicated that the periodontium region was intact in the Lig(−) group. The alveolar bone resorption, severe destruction of periodontal tissue, and inflammatory cell infiltration was obvious in the Lig(+)S‐PRG(−) group. However, in the Lig(+)S‐PRG(+) group, fewer inflammatory cells and less severe destruction of the collagen bundle and bone were observed in the periodontal tissue.

**Figure 5:**
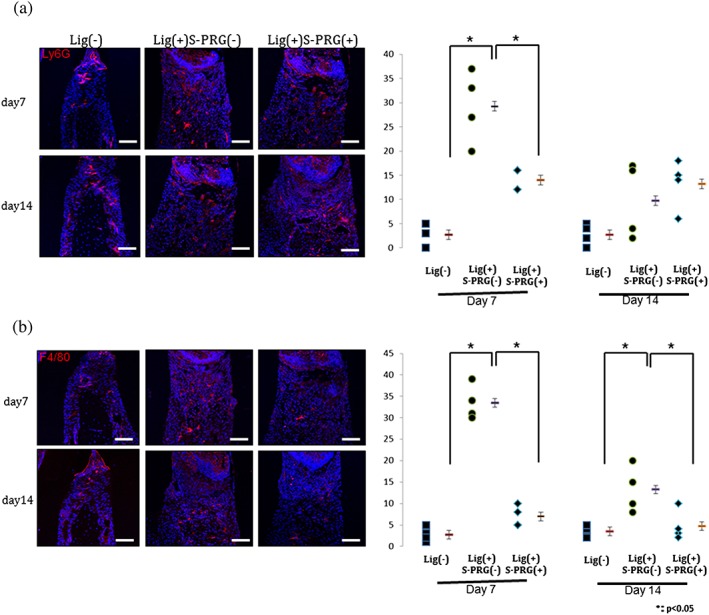
A representative image showing the immunohistochemical analysis of Ly6G‐positive neutrophils (a) and F4/80‐positive macrophages (b) in the cervical area on day 7 (upper) and day 14 (lower) (bar: 50 μm). The results of the quantitative analyses of the numbers of each cell type (/2500 μm^3^) are shown on the right

IHC analysis using Ly6G and F4/80 staining was performed to investigate the infiltration of neutrophils and macrophages, respectively, in the cervical area between the M1 distal root and the M2 mesial root (Figure 5). The infiltration of Ly6G‐positive neutrophils and F4/80‐positive macrophages was observed in the cervical area of the periodontal ligament on day 7, and the numbers of these cells were slightly decreased on day 14 in the Lig(+)S‐PRG(−) group. The quantitative analysis revealed that the numbers of Ly6G‐positive neutrophils and F4/80‐positive macrophages were decreased in the Lig(+)S‐PRG(+) group on day 7 compared with the Lig(+)S‐PRG(−) group. There was no significant difference between those numbers in the Lig(+)S‐PRG(+) group and the Lig(−) group.

### 3.3 Distribution of metal ions

**Figure 6:**
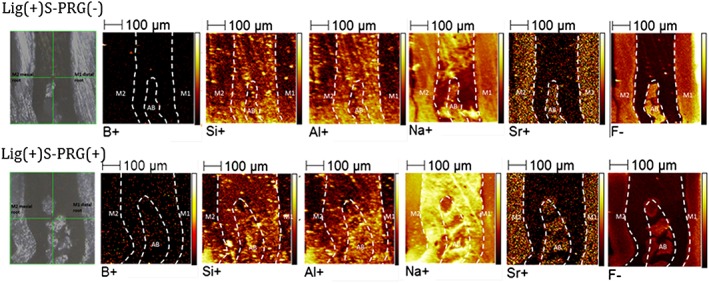
Representative time‐of‐flight secondary ion mass spectrometry images of six ions from the S‐PRG filler eluate between the M1 distal root and the M2 mesial root in the Lig(+)S‐PRG(−) and Lig(+)S‐PRG(+) groups

Because six metal ions released from the S‐PRG filler contributed to the prevention of periodontal tissue destruction, we next investigated whether these metal ions—boron, silica, aluminum, sodium, strontium, and fluoride—were incorporated into the periodontal tissue around the M2 mesial root using TOF‐SIMS analysis (Figure 6). To achieve this purpose, we investigated the M2 mesial root on day 14 to determine whether it was incorporated into the periodontal tissue of the ligature model. Strontium and fluoride ions were detected mainly in the hard tissue, including the alveolar bone and root dentin. By contrast, boron, aluminum, and silica ions were distributed throughout both the periodontal ligament and hard tissue. Among these ions, more boron ions were detected around the M2 mesial root in the Lig(+)S‐PRG(+) group than in the Lig(+)S‐PRG(−) group.

## 4 DISCUSSION

Periodontal disease is a prevalent oral inflammatory disease that leads to alveolar bone loss. Various animal models have been used to investigate host‐bacterial responses during the pathogenesis of periodontitis (de Molon et al., 2014; Dong, Huihui, & Li, 2015; Jiang et al., 2013; Yang, Zhang, Wang, Zhang, & Li, 2015). Recently, a mouse model of ligature‐induced periodontal disease was developed as a useful experimental model to investigate the molecular pathogenesis of periodontal disease, including the inflammatory response and the process of periodontal tissue destruction (Abe & Hajishengallis, 2013).

S‐PRG eluate has been reported to have antibacterial activity (Saku et al., 2010; Yoneda et al., 2012) and to inhibit oral biofilm formation *in vitro* (Shimazu et al., 2015; Suzuki et al., 2014). In this study, we applied the S‐PRG eluate to a ligature‐induced periodontal disease mouse model. The micro‐CT analysis showed that S‐PRG eluate significantly suppressed alveolar bone resorption in the ligature‐induced periodontal disease model. In addition, there was no significant difference in bone density between the Lig(−) group and Lig(+)S‐PRG(+) group. There is a possibility that metal ions of the S‐PRG eluate had some effect on bone density. Overall, the S‐PRG eluate inhibited alveolar bone loss in the ligature model both quantitatively and qualitatively.

The S‐PRG eluate also suppressed collagen destruction in the periodontal tissue and infiltration of inflammatory cells. (Hajishengallis & Sahingur, 2014) reported novel inflammatory pathways including Ly6G‐positive neutrophils in periodontitis. Yu et al. (2016) showed the F4/80 positive cells as total macrophages in periodontal infection. In this study, quantitative IHC analysis showed that the number of Ly6G‐positive neutrophils was significantly suppressed in the Lig(+)S‐PRG(+) group on day 7 compared with the Lig(+)S‐PRG(−) group. On the other hand, the number of F4/80‐positive macrophages was suppressed both on day 7 and day 14. In addition, there was no significant difference between the Lig(+)S‐PRG(+) group and the Lig(−)group. Because acute inflammation is characterized by emigration of primarily neutrophils, it is suggested that Ly6G‐positive cells were fewer on day 14. We showed for the first time that the S‐PRG eluate suppressed the destruction of periodontal tissue through its anti‐inflammatory effects *in vivo*.

It has been suggested that the combination of two or more ions has the potential to enhance bactericidal activity (Casemiro, Gomes Martins, Pires‐de‐Souza Fde, & Panzeri, 2008; Shinonaga & Arita, 2012). In the present study, we detected six ions around the M2 mesial root using TOF‐SIMS analysis and, in particular, showed that higher levels of boric ions were present in both the periodontal ligament and alveolar bone after treatment with S‐PRG eluate. Previously, the use of boric acid or chlorhexidine in periodontal pockets as an adjunct to non‐surgical periodontal treatment was shown to yield significant improvement over conventional treatment in clinical parameters (Saglam, Arslan, Buket Bozkurt, & Hakki, 2013). In rats, systemic administration of boric acid may reduce alveolar bone loss due to its interference with the RANKL/OPG balance in periodontal disease (Saglam, Hatipoglu, Koseoglu, Esen, & Kelebek, 2014). Our results suggest that metal ions released from S‐PRG filler might have the potential ability to exert anti‐inflammatory effects and, among metal ions, boric acid may play an important role in the prevention of ligature‐induced periodontal disease (Hakki, Bozkurt, & Hakki, 2010).

Prevention of alveolar bone loss is a key clinical challenge in the treatment of periodontal disease. Periodontal disease mainly occurs as a result of the activation of host‐derived immune and inflammatory mechanisms induced by cytokines, including IL‐1β, IL‐6, and TNF‐α (2009; 1996). IL‐1β and TNF‐α are proinflammatory cytokines that are involved in the initiation and progression of chronic inflammation, and they are critical mediators in the progression of periodontitis (Beklen et al., 2007; Graves & Cochran, 2003). As primary mediators, these cytokines not only promote neutrophil infiltration (Eskan et al., 2012) but also induce the production of other mediators, such as IL‐6, IL‐8, metalloproteinases, as well as prostaglandin E2, thereby facilitating and amplifying the inflammatory response in periodontal tissues (Graves & Cochran, 2003). These cytokines produce metalloproteinases, which degrade the extracellular matrix in the connective tissue, and RANKL, which initiates bone resorption. Thus, inhibition of the inflammatory cytokines could be a main target for the prevention of periodontal disease and tissue destruction. In the present study, we did not provide the precise mechanisms underlying S‐PRG eluate's ability to suppress alveolar bone resorption and infiltration of immune cells through the interference of cytokine activity.

Taken together, previous findings (Miki et al., 2016) and our data suggest that metal ions, such as boric acid, which are incorporated into the periodontal connective tissue, are capable of preventing the progression of bone resorption induced by inflammatory cytokines and that they play an important role in bone metabolism. We propose that the eluate of the S‐PRG filler could develop as a component of the oral rinsing solution as Suzuki et al. reported (Suzuki et al., 2014). To achieve these goals, further studies using the S‐PRG filler eluate should be performed to confirm its anti‐inflammatory properties and potential for development as a novel preventive treatment that modulates cytokine responses to suppress periodontal tissue destruction.

## CONFLICT OF INTEREST

None declared.

## Supporting information

Video S1. Supporting info item

Video S2. Supporting info item
